# Responses of leaf structure and photosynthetic properties to intra-canopy light gradients: a common garden test with four broadleaf deciduous angiosperm and seven evergreen conifer tree species

**DOI:** 10.1007/s00442-012-2279-y

**Published:** 2012-02-16

**Authors:** Tomasz P. Wyka, J. Oleksyn, R. Żytkowiak, P. Karolewski, A. M. Jagodziński, P. B. Reich

**Affiliations:** 1 Laboratory of General Botany, Institute of Experimental Biology, Department of Biology, Adam Mickiewicz University, Umultowska 89, 61-614 Poznań, Poland; 2 Institute of Dendrology, Polish Academy of Sciences, Parkowa 5, 62-035 Kórnik, Poland; 3 Department of Forest Resources, University of Minnesota, St. Paul, MN 55108 USA

**Keywords:** Plant functional types, Leaf plasticity, Shade acclimation, Evergreen leaves, Leaf mass-per-area

## Abstract

Spectra of leaf traits in northern temperate forest canopies reflect major differences in leaf longevity between evergreen conifers and deciduous broadleaf angiosperms, as well as plastic modifications caused by within-crown shading. We investigated (1) whether long-lived conifer leaves exhibit similar intra-canopy plasticity as short-lived broadleaves, and (2) whether global interspecific relationships between photosynthesis, nitrogen, and leaf structure identified for sun leaves adequately describe leaves differentiated in response to light gradients. We studied structural and photosynthetic properties of intra-tree sun and shade foliage in adult trees of seven conifer and four broadleaf angiosperm species in a common garden in Poland. Shade leaves exhibited lower leaf mass-per-area (LMA) than sun leaves; however, the relative difference was smaller in conifers than in broadleaves. In broadleaves, LMA was correlated with lamina thickness and tissue density, while in conifers, it was correlated with thickness but not density. In broadleaves, but not in conifers, reduction of lamina thickness was correlated with a thinner palisade layer. The more conservative adjustment of conifer leaves could result from a combination of phylogenetic constraints, contrasting leaf anatomies and shoot geometries, but also from functional requirements of long-lived foliage. Mass-based nitrogen concentration (N_mass_) was similar between sun and shade leaves, and was lower in conifers than in deciduous broadleaved species. Given this, the smaller LMA in shade corresponded with a lower area-based N concentration (N_area_). In evergreen conifers, LMA and N_area_ were less powerful predictors of area-based photosynthetic rate (*A*_max(area)_) in comparison with deciduous broadleaved angiosperms. Multiple regression for sun and shade leaves showed that, in each group, *A*_max(mass)_ was related to N_mass_ but not to LMA, whereas LMA became a significant codeterminant of *A*_max(mass)_ in analysis combining both groups. Thus, a fundamental mass-based relationship between photosynthesis, nitrogen, and leaf structure reported previously also exists in a dataset combining within-crown and across-functional type variation.

**Electronic supplementary material:**

The online version of this article (doi:10.1007/s00442-012-2279-y) contains supplementary material, which is available to authorized users.

## Introduction

Much effort in plant ecology has been devoted to identification of plant functional types, aiming to reduce the complexity of traits to a manageable level in order to enable realistic modeling of vegetation processes, vegetation-level photosynthesis, and productivity (Lavorel et al. 2007). Functional types have usually been defined on the basis of life history traits, plant habit, leaf and whole-plant longevity, and other easily measured traits. At least in some cases, correlations with other ecophysiological traits have been demonstrated, confirming the validity of such an approach (Reich et al. 1995, 1997, 2007; Wright et al. 2004, 2005a, b; Niinemets and Valladares 2006). In temperate forests, the two major and easily recognizable functional types of trees—and the subject of our investigation—are broadleaf deciduous angiosperms and evergreen conifers. The main functional basis for distinguishing the two groups is the difference in their leaf life-spans, although their separate phylogenetic histories underlie differences in other phenotypic features such as leaf structure, crown architecture, and wood composition.

Leaf life-span has been identified as a major co-variant of a number of ecophysiological traits. For example, longer leaf life-span is correlated with lower nitrogen concentration (Reich and Walters 1992; Reich et al. 1997; Wright et al. 2004), respiration rate (Reich et al. 1998), and maximal carboxylation rate (Wullschleger 1993). These differences appear to be strongly associated with structural traits of long-living leaves, such as higher LMA (Reich and Walters 1992; Reich et al. 1997; Wright et al. 2004), greater lamina thickness, content of mechanical tissues, and thickness and sclerification of mesophyll cell walls (Castro-Díez et al. 2000; Hanba et al. 2002), all likely resulting from the requirement to endure mechanical stress and herbivore pressure. Longer leaf life-span has also been associated with lower responsiveness of leaf traits to changes in environmental factors, such as level of nutrients (Aerts 1995) and light (Valladares et al. 2000; Wyka et al. 2007), but not CO_2_ (Tjoelker et al. 1998; Lee et al. 2001).

Light environment within a tree crown is heterogeneous due both to self-shading and to shading by neighboring trees. Leaves almost universally exhibit structural and functional plasticity in response to the crown light gradient (Ellsworth and Reich 1993; Hollinger 1996; Bond et al. 1999; Yoshimura 2010). However, the strength of these responses is species-specific (Sack et al. 2006) and may reflect an adaptive mechanism that could, among other possibilities, lead to optimization of whole plant gas exchange and resource investment strategy (Givnish 1988). Typically, sun leaves have higher LMA, are thicker, have a more pronounced palisade parenchyma, a greater area-based photosynthetic capacity (Givnish 1988), and shorter life-spans (Reich et al. 2004) compared to shade leaves from the same individual. So far, few ecological or life history predictors of the strength of the sun–shade leaf dichotomy have been identified in spite of an extensive research documenting its occurrence in various species (e.g., Strauss-Debenedetti and Berlyn 1994; Bond et al. 1999; Rozendaal et al. 2006; Oguchi et al. 2005; Sack et al. 2006). In particular, it is not clear how plant functional types differ with respect to the magnitude of these modifications. Especially, comparisons of reaction norms for structural and photosynthetic leaf traits of adult trees from our two focal groups grown under uniform conditions appear to be unavailable. Based on published studies (Givnish 2002; Valladares et al. 2000), it may be hypothesized that evergreen plants should exhibit lower trait plasticity than plants with short-lived foliage, although such a difference may not be universal (Markesteijn et al. 2007). Certainly, the developmental mechanisms and constraints underlying both structural and physiological adjustment to light levels may vary among species, functional types, and taxonomic groups. For example, the shade-induced reduction in thickness of palisade mesophyll is more pronounced in deciduous angiosperms than in evergreen conifers, at least in juvenile individuals (Youngblood and Ferguson 2003; Wyka et al. 2007).

In this paper, we studied adult, common garden-grown trees representing broadleaf deciduous angiosperm and evergreen conifer species in order to compare their abilities to adjust leaf structure and photosynthesis-related properties in response to intra-canopy light gradients. We tested the hypothesis that the former group exhibits a greater plasticity of the studied traits. Next, we examined the anatomical basis of structural adjustment in the two groups. We also tested the hypothesis that the two groups differ in the shapes of linear relationships linking structural, chemical, and photosynthetic traits in the manner predicted by local (Reich et al. 1995) and global analyses (Reich et al. 1998) when both high light (HL) and low light (LL) leaves are considered. Finally, we also tested whether these differences are a result of differences in both LMA and N concentrations, and thus disappear when photosynthesis is related to both of these traits simultaneously (Reich et al. 1998). The use of a ‘common garden’ experiment in this study provided an opportunity to minimize potential confounding effects of differences in climate, soil, topography, and land use. This allowed direct comparison of the effect of light conditions on studied leaf traits, even though it limited the sample size in each group.

## Materials and methods

### Study site and plant material

The trees used in this study included seven evergreen conifers: Macedonian pine (*Pinus peuce* Griseb.), noble fir (*Abies procera* Rehd.), Greek fir (*Abies cephalonica* Loundon), Douglas-fir (*Pseudotsuga menziesii* (Mirbel) Franco), grand fir (*Abies grandis* (Dougl. ex D. Don) Lindl.), Sawara false cypress (*Chamaecyparis pisifera* (Siebold & Zucc.) Endl.), and western red cedar (*Thuja plicata* Donn ex D. Don), and four broadleaf deciduous angiosperm species: northern red oak (*Quercus rubra* L.), yellow birch (*Betula alleghaniensis* Britton), red maple (*Acer rubrum* L.), and sugar maple (*Acer saccharum* Marsh.). These species represented a diverse range of geographic origins within the northern temperate climatic zones and have been introduced to Poland and tested at the field site for possible use as forest trees. All studied trees were 36–61 years old. They were growing in permanent, replicated single-species plots (typically ≈400 m^2^ each except for smaller plots for *Acer saccharum* and *Betula alleghaniensis*) at the Warsaw University of Life Sciences Arboretum in Rogów (51°48′N, 19°52′E, elevation 189 m a.s.l.; see Online Resources 1 and 2 for stand characteristics). Trees formed closed canopies, but at least some lateral branches at the edge of the plot were exposed to full sun. To characterize irradiance gradients within canopies, photosynthetic photon flux density (PPFD) was measured at four randomly selected locations in each plot at the lowest living branch (thus presumably representing the lowermost extreme of species’ shade tolerance), using PPFD sensors (Li1000; LiCor, Lincoln, NE, USA). Simultaneously, PPFD was determined in an adjacent, fully exposed location to provide reference irradiance level for calculation of relative irradiance. Measurements were conducted on an overcast day and thus provide a reliable estimate of average light conditions during the “in-leaf” growing season (Tobin and Reich 2009). Relative irradiances at the lowest living branch were below 10% of ambient light in all species, with the lowest values noted in *Thuja plicata* (1.5%) and in the two *Acer* (below 3%; Online Resource 1).

### Light-saturated net photosynthesis (*A*_max_) measurements

Four individual trees per species were chosen for study (usually 2 from each of the 2 plots on which a species was growing), with additional trees selected in cases when a single tree did not provide access to both fully shaded and fully illuminated leaves. Shoots for measurements were selected from a fully sun exposed (high light, HL) and the lowermost living (low light, LL) branch. Leaves from such locations thus represented the extreme expression of HL and LL syndromes in each species. Shoots (approximately 50–80 cm long) were cut using pole pruners shortly prior to measurement. All shoots originated from height not greater than 8 m. The cut ends were placed in water and re-cut. Prior tests of field net CO_2_ exchange rates versus those measured on detached shoots showed no significant differences (Ellsworth and Reich 1993). To ensure full photosynthetic activation, HL shoots were maintained in a fully sunlit spot, whereas LL shoots were kept under PPFD around 500 μmol m^−2^ s^−1^. In cloudy weather, shoots were given supplementary halogen illumination up to c. 500 μmol m^−2^ s^−1^. *A*_max_ measurements were conducted on site within 2 h after shoot harvest using Li-6400 gas exchange system (LiCor) operating in an open mode. Shoot collection and measurements were conducted from mid-morning to mid-afternoon as no persistent midday decline of stomatal conductance and no temporal trends in photosynthetic rate were observed. For broadleaves and *Pinus* needles, we used the broadleaf chamber fitted with a LED light source (PPFD = 1,500 μmol m^−2^ s^−1^), whereas for conifers, we used the conifer chamber and an external halogen lamp providing at least 600 μmol m^−2^ s^−1^ quanta. We performed light response curves (using PPFD up to 1,500 μmol m^−2^ s^−1^ quanta) on three species from each group, and found that such light levels caused saturation of photosynthesis and that no inhibition of photosynthesis occurred. Leaf temperature during measurement was maintained at 22–26°C with an occasional rise to 28°C which did not appear to influence the photosynthetic rates. Relative humidity in the chamber during the measurement was 50–70%. Leaves used for *A*_max_ measurements were analyzed for carbohydrate and N concentration. Immediately after collection, leaves were placed in an ice-box and transported to the laboratory where they were dried at 65°C for 48 h. Four replicate *A*_max_ measurements were made for each species, and light conditions.

### Chemical analyses

Oven-dried leaf tissue was pulverized in Culatti Mikro-Feinmühle (IKE Labortechnik Staufen, Germany). Concentration of total nonstructural carbohydrates was measured colorimetrically as described previously (Oleksyn et al. 2000). Carbohydrate-free leaf dry mass was calculated and used as a basis for calculation of leaf variables.

For determination of nitrogen concentration, leaf tissue samples were subjected to analysis in an Elemental Combustion System CHNS-O 4010 (Costech Instruments, Italy/USA).

### Leaf structure and anatomy

For determination of leaf mass per area (LMA) in broadleaved species, the 2 × 3 cm leaf segment used for photosynthesis measurement was carefully excised, oven-dried and weighed. In conifers, leaves were plucked from the twig, transported to the laboratory, scanned for total area using WinSeedle Software (Regent Instruments, Quebec, Canada), dried and weighed. Entire twigs were scanned in cases of *Chamaecyparis pisifera* and *Thuja plicata*.

For anatomical studies, small leaf fragments were fixed overnight at 4°C in a solution consisting of 2% glutaraldehyde and 2% paraformaldehyde in cacodylate buffer (pH 7.0). In case of broadleaves, samples were dehydrated in a graded ethanol series from 10 to 100% ethanol, followed by butanol. Afterwards, they were embedded in Paraplast Plus (Sigma, Saint-Louis, MO, USA) and sectioned with a microtome, followed by staining in safranine and fast green. Conifer leaf samples were passed through series of ethanol solutions (up to 70%), then immobilized in styrofoam blocks, hand-cut with a razor blade, and stained with floroglucine. Care was taken to obtain sections perpendicular to leaf surface. Sections were examined through a light microscope (Axioskop; Carl Zeiss, Oberkochen, Germany) and photographed using an attached Powershot G5 camera (Canon, Tokyo, Japan). Measurements were taken from digital images using LSM 510 Image Browser software (Carl-Zeiss, Göttingen, Germany). For each leaf section, lamina thickness at inter-veinal location, mesophyll thickness, palisade thickness, and length of longest cells in the outermost adaxial layer were determined (these measurements in conifer needles were taken midway between the edge and the central vein except in *P. peuce*, where they were taken along central axis of the needle section). Since palisade in conifers was not always well defined, we considered this outermost layer as representing palisade and also included underlying cells if their length was at least twice as large as the width. To determine leaf density (LD, g cm^−3^) while accounting for unevenly thickened leaf samples, leaf volume-per-area, an integrated measure of leaf thickness (LVA, cm^3^ m^−2^) was first estimated from microscopic sections (where LVA was taken as section area × section width^−1^). Leaf tissue density was then calculated as LD = LMA × LVA^−1^ according to Poorter et al. (2009), using average LMA values for each species and light conditions. Four leaves per species from each light environment were sampled. For each leaf, three sections were used for measurements and results were averaged to obtain independent data points.

### Statistics

Variables were routinely log_10_-transformed to homogenize variances (other transformations were occasionally used for ratio variables). Two-way analysis of variance was used to test the effects of species and light environment on the various variables. ANOVAs were run separately for broadleaves and conifers, and also for the entire set of species. These analyses were followed by pair-wise contrasts between shaded and fully exposed leaves within each species. Additionally, plasticity indices [PI = (min−max)/max, where min and max are mean minimal and maximal value for each trait] were calculated for all variables in each species (Valladares et al. 2000). To directly compare broadleaves and conifers, another set of two-way ANOVAs was run to test the effects of functional type and light, followed by Tukey’s test. Correlations were calculated and least square regression lines were fitted to log_10_ transformed variables to test the influence of variables that were considered to represent primary responses on those deemed to be derived. Since anatomical measurements and LMA determination were not conducted on the same (but rather on neighboring) leaves, we used species means to study the correlation of LMA and leaf thickness. Analysis of covariance was used to test for differences in slopes of regression lines between groups. To test whether in a dataset combining HL and LL data, nitrogen concentration, and LMA provide sufficient information to predict *A*_*max*_, as previously shown for HL leaves (Reich et al. 1998; Wright et al. 2004), a multiple linear regression model was fitted to the pooled dataset as well as separately to the two groups. We focused on mass-based measures of N and photosynthesis because (unlike in area-based measures) there was no within-group correlation between N_mass_ and LMA (each *P* > 0.05), thus providing independent explanatory variables. All statistical analyses were conducted using Statistica software (Statsoft, Tulsa, OK, USA).

## Results

### Structural traits

**Fig. 1:**
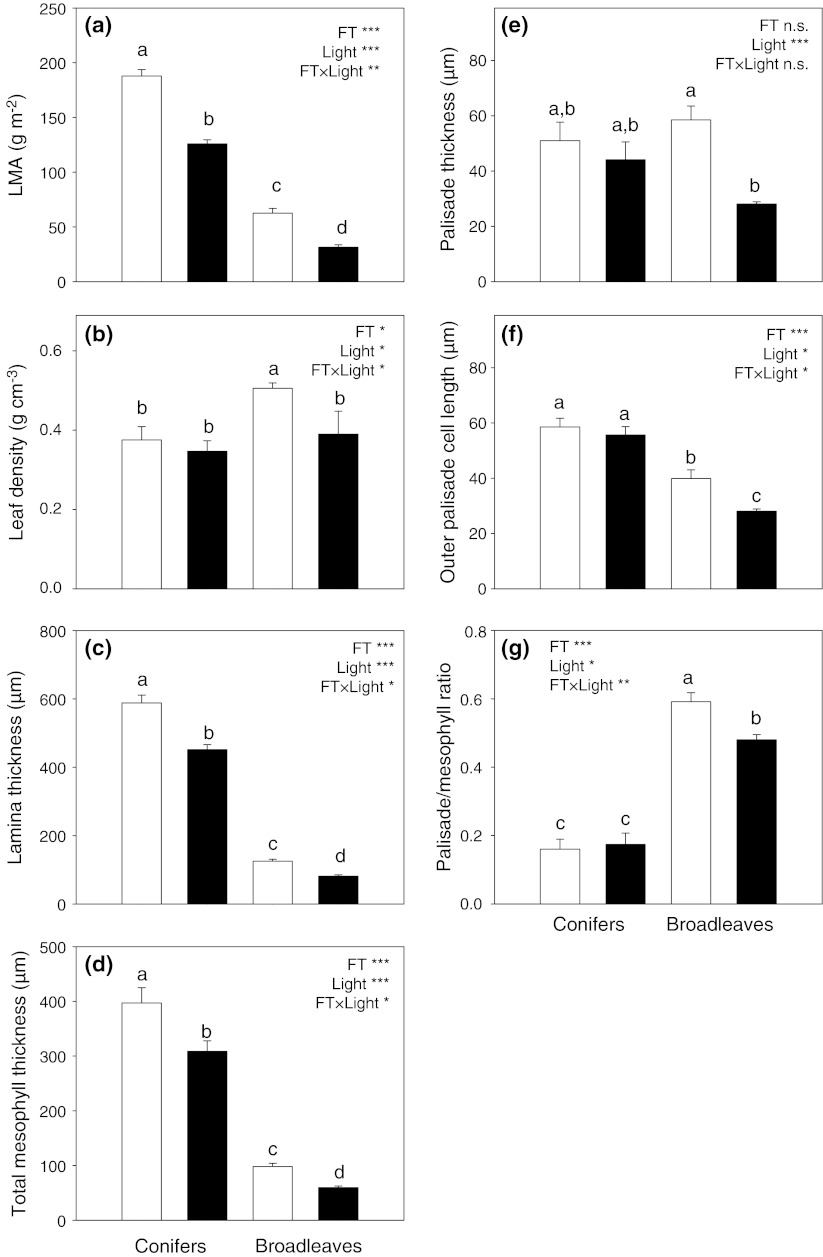
Values of structural traits (means ± SE) in sun leaves (*open bars*) and shade leaves (*filled bars*) of seven evergreen conifer and four deciduous broadleaf tree species averaged within the functional types. Results of ANOVA are shown (*FT* functional type). *Shared letters* indicate lack of a significant difference in pairwise comparisons by Tukey’s test. *Asterisks* indicate significant ANOVA effects (*P < 0.05, **P < 0.01, ***P < 0.001), *n.s.* effect not significant

**Fig. 2:**
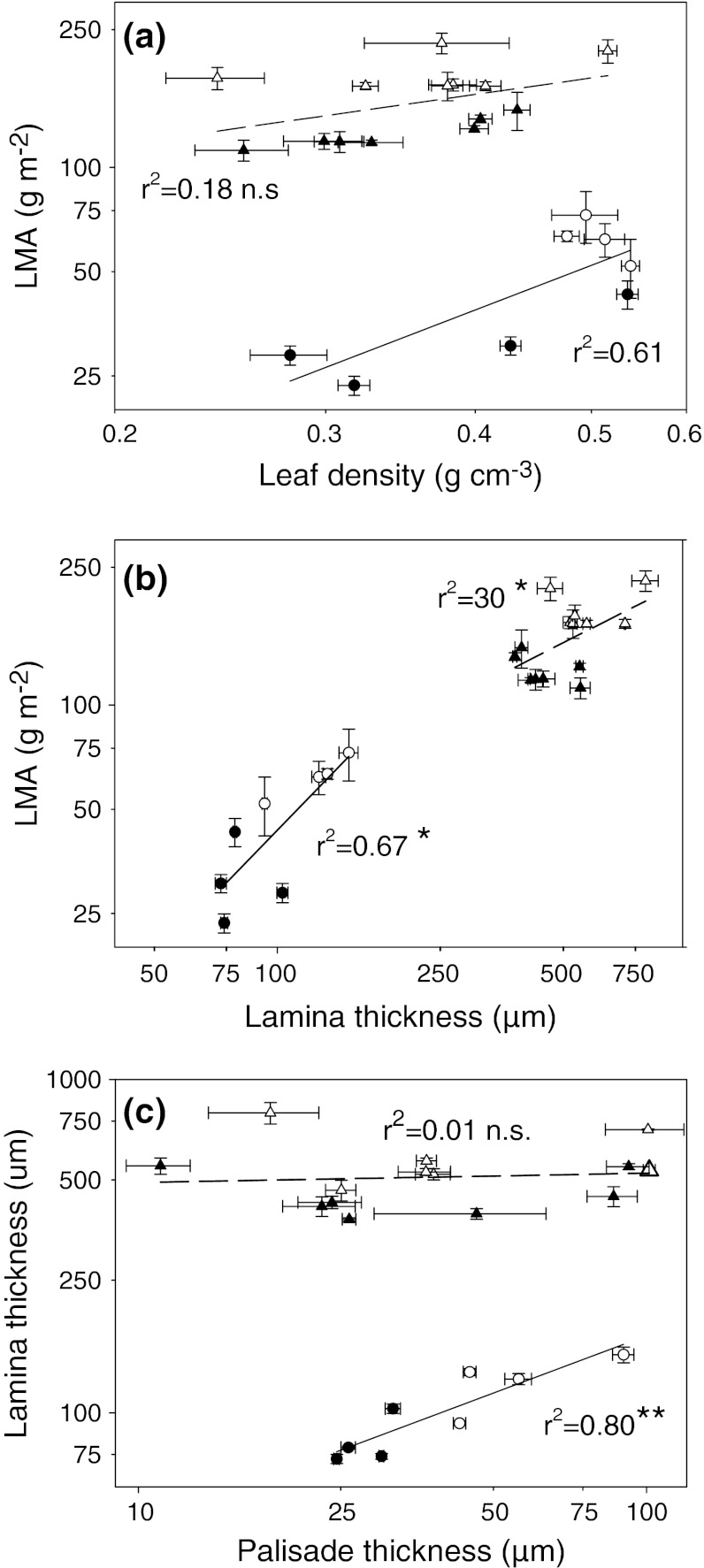
Relationships between average (±SE) values of **a** leaf density and LMA, **b** leaf lamina thickness and LMA, and **c** palisade thickness and lamina thickness in seven conifer (*triangles*) and four broadleaf (*circles*) tree species. Sun leaves are marked by *open symbols* and shade leaves by *filled symbols*. Note log axes. Regression coefficients are shown. Linear regression equations for conifers and broadleaves are, respectively: **a** log(LMA) = 2.40 + 0.48 × log(leaf density) (*r* ^2^ = 0.12, *P* > 0.05, *n* = 14) and log(LMA) = 2.08 + 1.26 × log(leaf density) (*r* ^2^ = 0.61, *P* < 0.05, *n* = 8), Ancova functional type × log(leaf density) *P* = 0.12; **b** log(LMA) = 0.55 + 0.60 × log(lamina thickness) (*r* ^2^ = 0.30, *P* < 0.05, *n* = 14) and log(LMA) = −0.77 + 1.20 × log(lamina thickness) (*r* ^2^ = 0.67, *P* < 0.05, *n* = 8), Ancova functional type × log(blade thickness) *P* = 0.19 Ancova functional type × log(palisade thickness) *P* < 0.001; **c** log(lamina thickness) = 2.66 + 0.33 × log(palisade thickness) (*r* ^2^ = 0.01, *P* < 0.76, *n* = 14) and log(lamina thickness) = 1.09 + 0.57 × log(palisade thickness) (*r* ^2^ = 0.80, *P* < 0.01, *n* = 8) Ancova functional type × log(palisade thickness) *P* < 0.01

Significant interspecific variation was found for all structural traits when analyzed separately for each functional type or across the whole dataset (trait values and statistics are presented in Online Resource 3). Light level affected all structural traits in broadleaves and, in most cases, in conifers. As expected, sun leaves of all species had significantly greater LMA than did shade leaves (Fig. 1a). LMA in broadleaves was typically about 50% lower in LL than in HL leaves, whereas in conifers, this difference was on average smaller (as indicated by significant interaction term in Fig. 1a), and ranged between 20% in *A. cephalonica* and 51% in *C. pisifera* (Table 1). Although light level significantly affected leaf tissue density, there were also significant interactions with species. Whereas in broadleaves, in three out of four species leaf density was lower in LL, in conifers, the pattern was absent (Fig. 1b; Online Resource 3). In conifers, LMA was not related to leaf density, while in broadleaves, the correlation was positive and significant (Fig. 2a). In both groups, sun leaves were thicker than shade leaves and had thicker mesophyll (Fig. 1c, d; Table 1; Online Resource 2). In both groups, lamina thickness was a significant correlate of LMA across all species and light levels, with similar slopes but a stronger determination in the broadleaf trees (*r* ^2^ = 0.67, *n* = 8, *P* < 0.05) than in conifers (*r* ^2^ = 0.30, *n* = 14, *P* < 0.05; Fig. 2b). Together, these results show that the lower LMA in LL leaves in broadleaves was associated with consistently smaller leaf density and thickness, whereas in conifers, the mechanism of LMA adjustment was species-specific, typically involving a decrease in thickness but only seldom a lowering of leaf density.

**Table 1:** Plasticity indices (PI) of structural and photosynthetic leaf traits of evergreen conifer and deciduous broadleaf trees calculated for sun versus shade leaves based on data from Online Resource 1

Trait	Gymnosperms							Angiosperms			
	*Pinus peuce*	*Abies cephalonica*	*Pseudotsuga menziesii*	*Abies grandis*	*Thuja plicata*	*Abies procera*	*Chamaecyparis pisifera*	*Acer sacharum*	*Acer rubrum*	*Betula alleghanensis*	*Quercus rubra*
Structural											
LMA	**0.25**	**0.20**	**0.31**	**0.32**	**0.34**	**0.33**	**0.51**	**0.55**	**0.51**	**0.55**	**0.41**
Leaf tissue density	0.02	**0.20**	0.14	**0.16**	**0.20**	**0.32**	**0.19**	**0.41**	**0.17**	**0.41**	0.08
Lamina thickness	**0.23**	**0.33**	**0.21**	**0.18**	**0.16**	**0.15**	**0.31**	**0.20**	**0.42**	**0.22**	**0.47**
Mesophyll thickness	**0.28**	**0.32**	**0.18**	**0.19**	**0.16**	**0.20**	**0.28**	**0.21**	**0.47**	**0.23**	**0.57**
Palisade thickness	0.08	**0.30**	**0.38**	**0.37**	0.15	**0.46**	**0.39**	**0.30**	**0.56**	**0.29**	**0.71**
Outer palisade cell length	0.14	**0.26**	0.20	0.08	0.06	0.09	0.15	**0.30**	**0.56**	**0.28**	**0.32**
Palisade/mesophyll ratio	**0.23**	0.04	0.24	0.21	0.01	**0.53**	0.27	**0.11**	**0.17**	0.08	**0.34**
Photosynthetic											
N_area_	0.19	0.14	**0.36**	**0.34**	**0.39**	**0.48**	**0.41**	**0.47**	**0.49**	**0.58**	**0.50**
N_mass_	0.09	0.09	0.03	0.03	0.05	0.18	**0.23**	0.12	0.05	0.10	0.14
*A*_max(area)_	**0.15**	**0.37**	0.19	0.23	**0.51**	**0.55**	**0.60**	0.29	**0.54**	**0.63**	**0.49**
*A*_max(mass)_	0.12	0.22	0.16	0.12	0.24	0.26	0.17	**0.36**	0.12	0.19	0.19
PNUE	0.07	0.23	0.20	0.15	0.19	0.11	0.33	**0.27**	0.09	0.11	0.03

Bold font indicates significant contrasts between the two types of leaves

In all species (except *Pinus peuce*), the mesophyll contained at least a single layer of distinct palisade cells. In *P. peuce*, the outer layer of roughly isodiametric cells was, for comparative purposes, considered to represent the palisade. The palisade layer was thicker in sun leaves and the difference between sun and shade leaves was relatively greater in broadleaves (plasticity index up to 71% in *Quercus rubra*; Table 1; Fig. 1e). Among the conifers, the extent of palisade development did not differ between sun and shade leaves in *Pinus peuce* and *Thuja plicata*, and was greater in shade than in sun leaves in *Abies procera* (Table 1; Online Resource 3). As a consequence, the correlation between palisade and lamina thickness was significant only in broadleaves (*r* ^2^ = 0.80, *P* < 0.01, *n* = 32; Fig. 2c). When length of the outermost adaxial cells alone (i.e. the upper palisade layer) was considered, most conifers did not show significant differences between sun and shade leaves, whereas among broadleaves, these cells were longer in sun leaves in three out of the four species (Fig. 1f; Table 1; Online Resource 3). The contribution of palisade layer to mesophyll thickness was greater in broadleaves, where palisade accounted for about half of mesophyll thickness, compared to conifers, where it typically did not exceed 20%. A notable exception was *Pinus peuce*, a species with large cells arranged in few layers. The palisade/mesophyll ratio was greater in sun than in shade leaves of most broadleaves but showed no clear trend (and usually no significant HL vs. LL differences) in conifers (Fig. 1g; Table 1). As seen from Fig. 1 and Table 1, structural variability of leaves within the crown in broadleaf deciduous angiosperms was relatively larger and more predictable than in evergreen conifers.

### Nitrogen concentrations

**Fig. 3:**
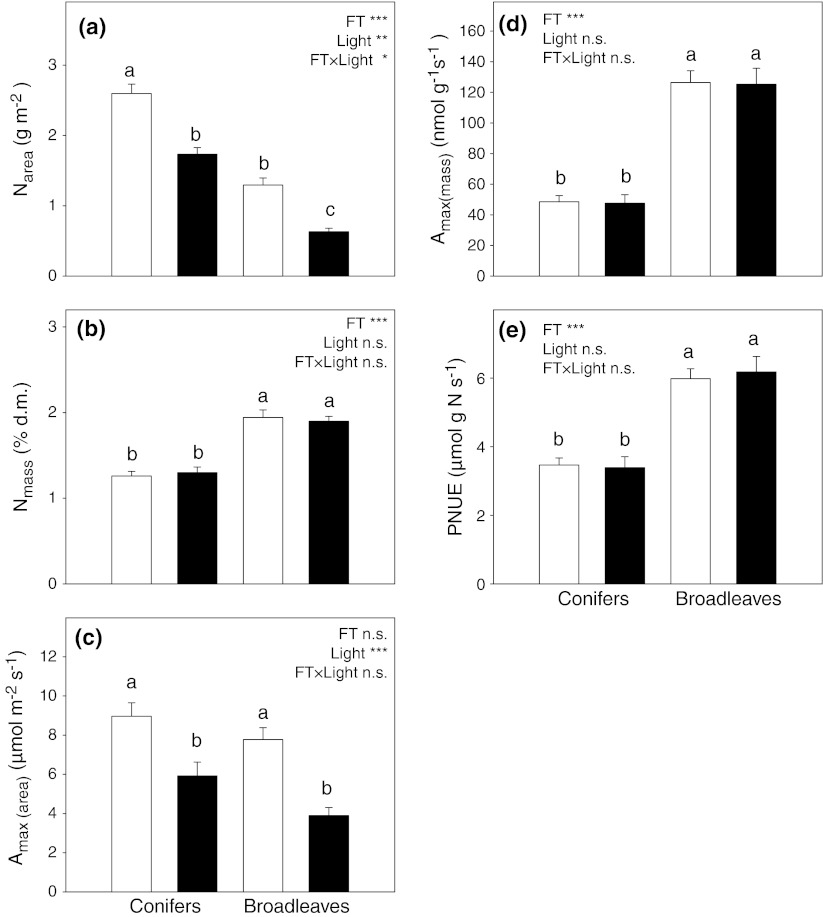
Photosynthetic traits (means ± SE) in sun leaves (*open bars*) and shade leaves (*filled bars*) of seven evergreen conifer and four deciduous broadleaf angiosperm tree species averaged within the functional types. Results of ANOVA are shown. See legend to Fig. 1 for explanation of symbols and abbreviations

**Fig. 4:**
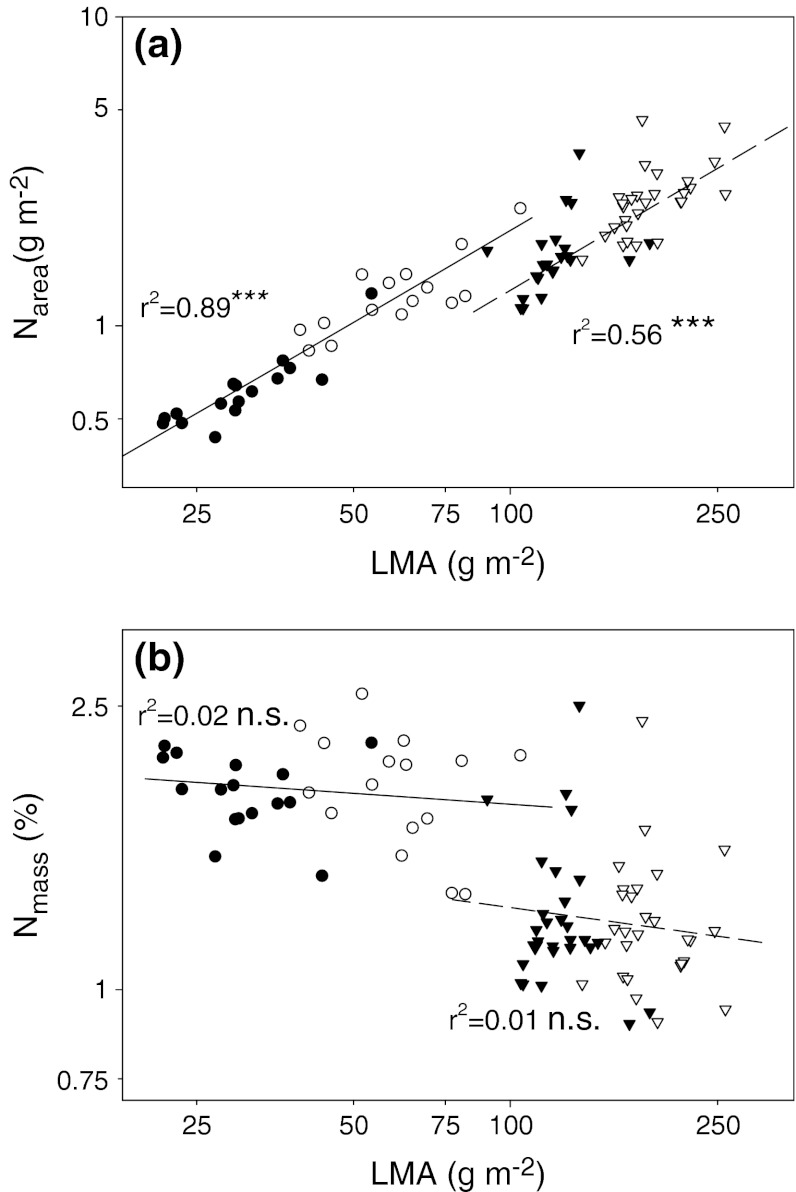
Relationships between N concentration and LMA on **a** leaf area and **b** leaf mass basis. For explanation of symbols, see legend to Fig. 2. Regression equations are:** a** conifers log(N_area_) = −1.76 + 0.95 × log(LMA), *n* = 56, *r* ^2^ = 0.56, *P* < 0.001; broadleaves log(N_area_) = −1.63 + 0.97 × log(LMA), *n* = 32, *r* ^2^ = 0.89, *P* < 0.001; Ancova functional type × log(LMA) term *P* = 0.91.** b** Conifers log(N_mass_) = 0.32 − 0.10 × log(LMA), *n* = 56, *r* ^2^ = 0.01, *P* > 0.05; broadleaves log(N_mass_) = 0.37 − 0.05 × log(LMA), *n* = 32, *r* ^2^ = 0.02, *P* > 0.05

Nitrogen concentration on an area basis was lower in broadleaves than in conifers, and it was significantly lower in shade in both functional groups and in almost all species (Table 1; Fig. 3a; Online Resource 3). On a mass basis, nitrogen concentration was larger in broadleaves but did not differ between sun and shade leaves in either group (Table 1; Fig. 3b). The relationships of N_area_ to LMA were positive and the slopes did not differ between broadleaves and conifers (Ancova interaction term *P* = 0.91; Fig. 4a). However, the lower intercept in conifers indicated that, at comparable LMA, a conifer leaf would contain less N_area_. On the other hand, there were no significant within-group relationships between N_mass_ and LMA (Fig. 4b). Thus, although broadleaves had on average greater N_mass_ than conifers, relatively larger between-group differences in LMA resulted in conifer leaves containing more nitrogen per area than broadleaves at both ends of the light gradient (Figs. 3a, 4a).

### Maximal photosynthetic rate

Consistent with similar N_mass_ values, photosynthetic rates expressed per unit mass were not significantly affected by growth irradiance (except for *Acer saccharinum* where shade leaves showed higher photosynthesis (Table 1; Online Resource 3). Hence, again as a result of differences in LMA (and therefore in N_area_), shade leaves of all species showed smaller maximal area-based photosynthetic rates compared to sun leaves, although the difference was not significant in three species (Table 1). The extent of reduction ranged widely (from 15% in *Pinus peuce* to 63% in *Betula alleghanesis*), but was not significantly different between broadleaf and conifer trees (Fig. 3c). As noted previously (Reich and Walters 1992; Reich et al. 1995), *A*_max_ expressed per unit of leaf mass or leaf N was much greater in deciduous broadleaf angiosperms than in evergreen conifers (both for sun and shade leaves; Fig. 3d, e).

**Fig. 5:**
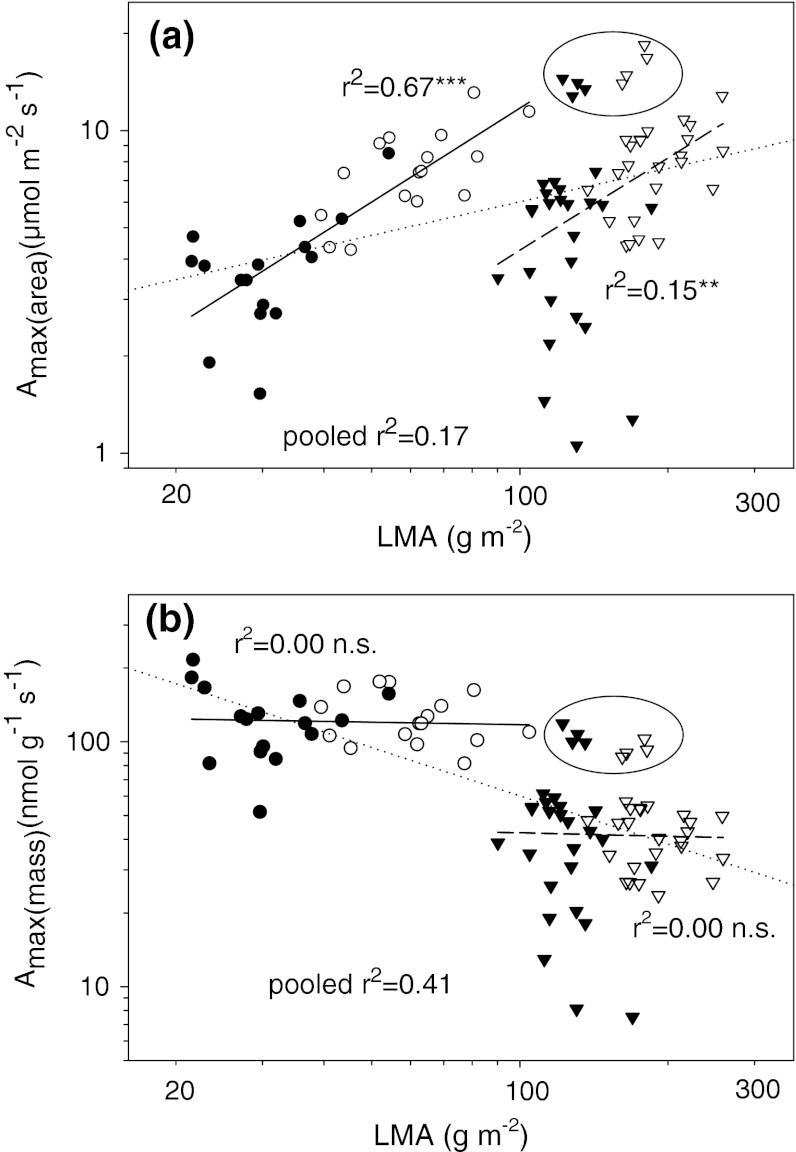
Relationships between leaf mass-per-area (LMA) and **a** area-based maximal photosynthetic rate (*A*_max(area)_) and **b** mass-based maximal photosynthetic rate (*A*_max(area)_). *Dotted lines* mark regression for pooled data. For explanation of other symbols, see legend to Fig. 2. *Ellipses* contain data points for *Pinus peuce*. Regression equations are: **a** conifers log(*A*_max(area)_) = −1.28 + 0.95 × log(LMA), *n* = 56, *r* ^2^ = 0.15, *P* < 0.001; broadleaves log(*A*_max(area)_) = −0.86 + 0.96 × log(LMA), *n* = 32, *r* ^2^ = 0.67, *P* < 0.01; pooled data log(*A*_max(area)_) = 0.09 + 0.34 × log(LMA), *n* = 87, *r* ^2^ = 0.16, *P* < 0.001; **b** conifers log(*A*_max(mass)_) = 1.72 − 0.05 × log(LMA), *n* = 56, *r* ^2^ = 0.00, *P* > 0.05; broadleaves log(*A*_max(mass)_) = 2.14 − 0.03 × log(LMA), *n* = 32, *r* ^2^ = 0.67, *P* > 0.05; pooled data log(*A*_max(mass)_) = 3.10 − 0.66 × log(LMA), *n* = 87, *r* ^2^ = 0.42, *P* < 0.001. Ancova functional type × log(LMA) terms *P* > 0.05 in both panels

*A*_max(area)_ was positively related to LMA, especially in broadleaves, where the slope was similar but the determination coefficient larger (slope = 0.97, *r* ^2^ = 0.67, *n* = 32, *P* < 0.001) in comparison to conifers (slope = 0.95, *r* ^2^ = 0.15, *n* = 56, *P* < 0.001; Fig. 5a). Our data did not reveal any significant correlation between *A*_max(mass)_ and LMA in either group (Fig. 5b). However, for pooled samples the relationship of *A*_max(area)_ to LMA was weaker (*r* ^2^ = 0.17, *n* = 88, *P* < 0.001) than the relationship of *A*_max(mass)_ to LMA (*r* ^2^ = 0.42, *n* = 88, *P* < 0.001), highlighting functional-type specific scaling of the former and a cross-type convergence of the latter (see legend to Fig. 5 for equations).

**Fig. 6:**
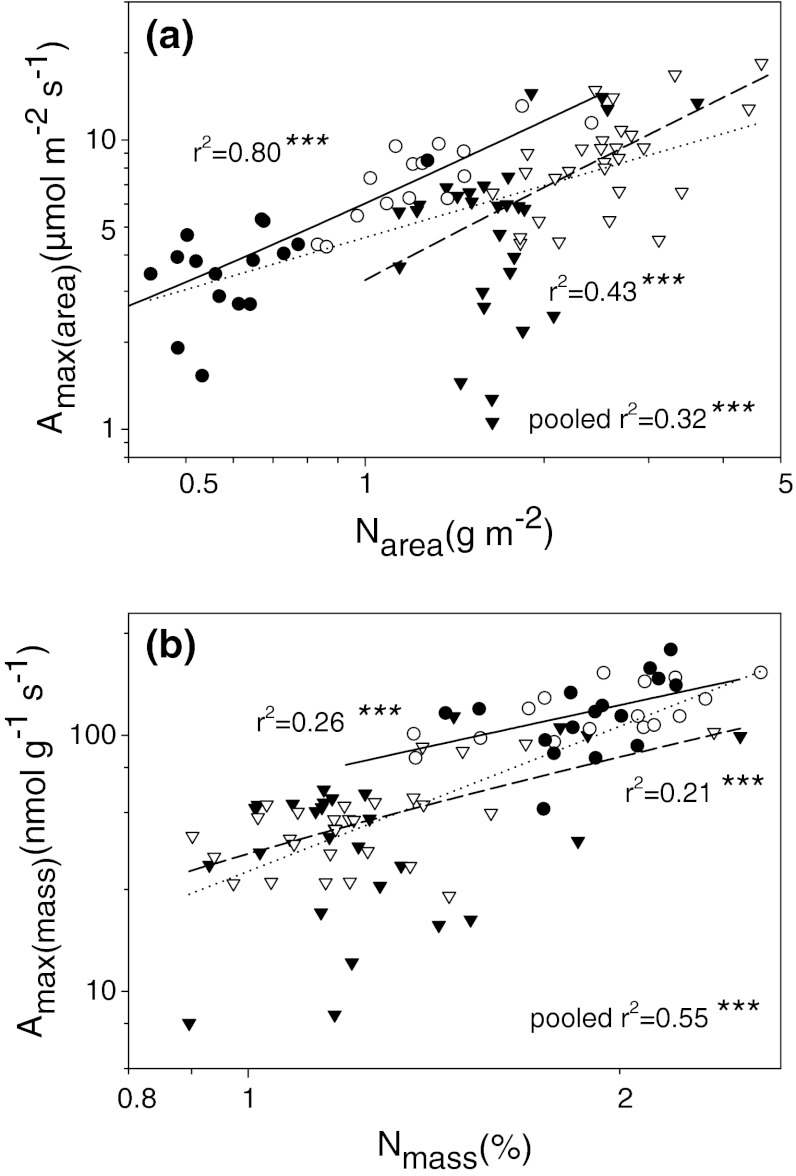
Area-based (**a**) and mass-based (**b**) relationships between N and *A*_max_. *Dotted lines* mark regression for pooled data. For explanation of other symbols, see legend to Fig. 2. Regression equations are: **a** conifers log(*A*_max(area)_) = 0.46 + 1.10 × log(N_area_), *r* ^2^ = 0.32, *n* = 56, *P* < 0.001; broadleaves log(*A*_max(area)_) = 0.77 + 1.00 × log(N_area_), *r* ^2^ = 0.75, *n* = 32, *P* < 0.001, pooled data log(*A*_max(area)_) = 0.66 + 0.60 × log(N_area_), *r* ^2^ = 0.32, *n* = 88, *P* < 0.001, Ancova functional type × log(N_mass_) *P* > 0.05; **b** conifers log(*A*_max(mass)_) = 3.46 + 1.21 × log(N_mass_), *r* ^2^ = 0.21, *n* = 56, *P* < 0.001; broadleaves log(*A*_max(mass)_) = 4.17 + 0.97 × log(N_mass_), *r* ^2^ = 0.26, *n* = 32, *P* < 0.01, pooled data log(*A*_max(mass)_) = 3.41 + 1.88 × log(N_mass_), *r* ^2^ = 0.55, *n* = 88, *P* < 0.001, Ancova functional type × log(N_mass_) *P* > 0.05

**Fig. 7:**
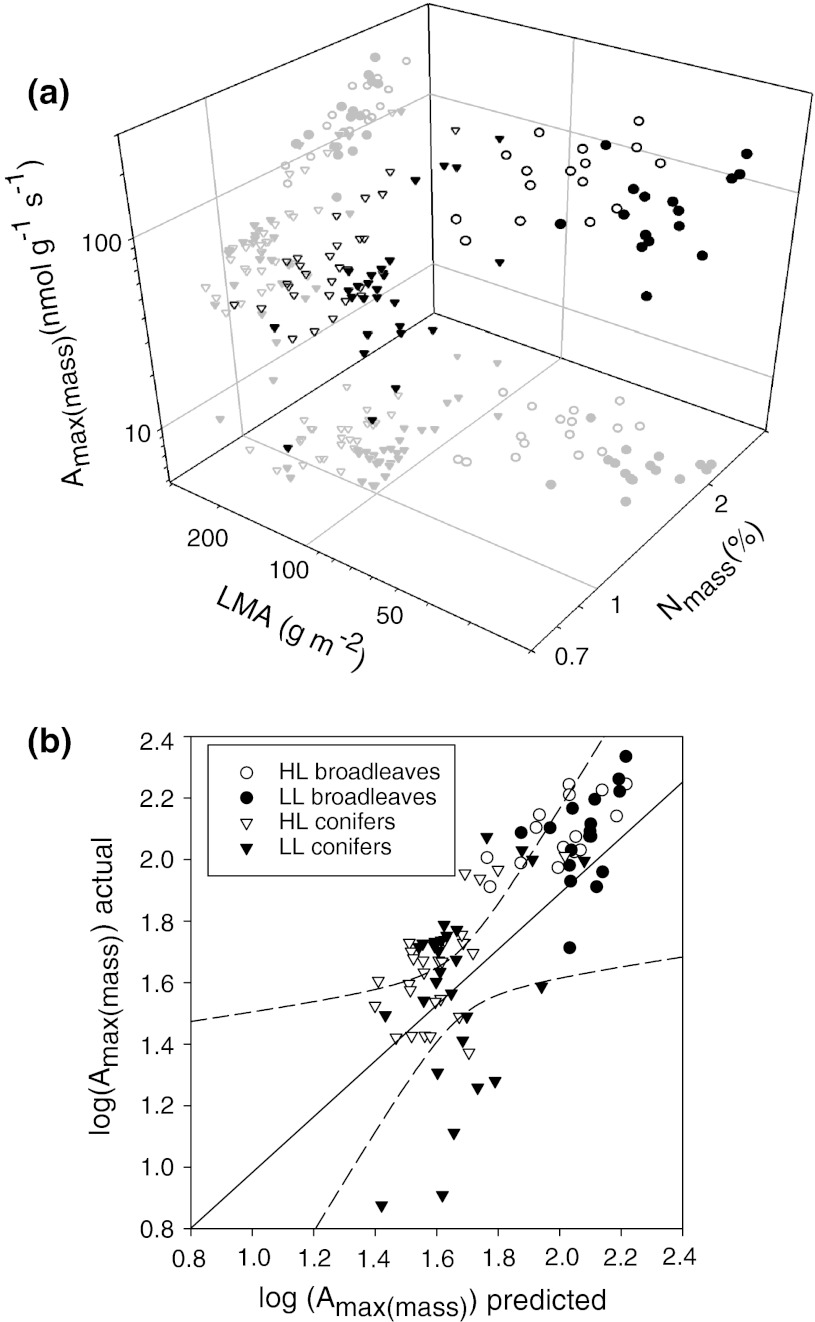
**a** Relationship of *A*_max(mass)_ to N_mass_ and LMA for conifer and broadleaf samples including HL and LL leaves. Regression equations are: for conifers log(*A*_max(mass)_) = 1.33 + 1.22 × log(N_mass_) + 0.08 × log(LMA), *r* ^2^ = 0.21, *P* < 0.002, *n* = 56; broadleaves log(*A*_max(mass)_) = 1.77 + 0.98 × log(N_mass_) + 0.02 × log(LMA), *r* ^2^ = 0.28, *P* < 0.001, *n* = 32 and pooled data log(*A*_max(mass)_) = 2.09 + 1.42 × log(N_mass_) − 0.27 × log(LMA), *r* ^2^ = 0.60, *P* < 0.001, *n* = 88. **b** Actual versus predicted plot of log_10_ transformed *A*_max(mass)_ for the pooled regression from (**a**). *Dashed lines* indicate 95% confidence intervals

In both groups, *A*_max_ was correlated with N on both area and mass basis (Fig. 6), and the regression slopes were statistically similar in conifers and in broadleaves (area-based slope = 1.10, *r* ^2^ = 0.21, *n* = 56, *P* < 0.001 vs. 1.00 *r* ^2^ = 0.77, *n* = 32, *P* < 0.001; mass-based slope = 1.21, *r* ^2^ = 0.21, *n* = 56, *P* < 0.001 vs. 0.97, *r* ^2^ = 0.26, *n* = 32, *P* < 0.01; in both cases, Ancova functional type × log(N_mass_) *P* > 0.05). When data were pooled across groups, the relationship became stronger on mass basis (*r* ^2^ = 0.55, *n* = 88, *P* < 0.001) but not on area basis (*r* ^2^ = 0.32, *n* = 88, *P* < 0.001). Since *A*_max(mass)_ was significantly correlated to both LMA and N_mass_ across functional types, a multiple regression model was fitted to the whole dataset, without regard to the affinity of samples (Fig. 7). This yielded a significant relationship (*r* ^2^ = 0.60, *P* < 0.001) that confirmed contributions of both N_mass_ (slope = 1.42, *P* < 0.001) and LMA (slope = −0.27, *P* < 0.01). The LMA × N_mass_ interaction term, when included, was not significant and did not improve the fit (not shown). Multiple regression model fitted to conifer and broadleaf samples separately confirmed the positive relationship of *A*_max(mass)_ with N_mass_ (partial slopes significant at *P* < 0.001 and *P* < 0.01, respectively) and lack of relationship with LMA (*P* > 0.5 in each case; see legend to Fig. 7 for equations) as noted in simple regression. The importance of LMA was therefore only seen in a cross-functional type comparison. To evaluate the influence of LL data on the strength of relationships, LL data points were deleted from the above models. When multiple regression analysis was run for HL leaves alone the overall determination coefficient increased for the pooled data (from *r* ^2^ = 0.60 to *r* ^2^ = 0.78, *n* = 44, *P* < 0.001) as well as for functional types (conifers from *r* ^2^ = 0.21 to *r* ^2^ = 0.37, *n* = 28, *P* < 0.01, broadleaves from *r* ^2^ = 0.26 to *r* ^2^ = 0.43, *n* = 16, *P* < 0.05) in spite of smaller number of data points, suggesting a tendency for LL leaves, especially in conifers to deviate from the general trends (Fig. 7b).

## Discussion

By studying leaves from extreme ends of intracanopy light gradients in evergreen conifer and broadleaf deciduous angiosperm trees, we found that the acclimation capacity to reduced light consistently differs between these groups with respect to several important structural and chemical traits influencing leaf photosynthetic potential. We consider the trait spectra measured here to represent the actual reaction norms of adult individuals, as the LL branches were sampled from the lowermost, i.e. most shaded, positions in the closed canopy. Structural diversification in response to different light availability was smaller in leaves of evergreen conifers as also noted in previous studies on conifers (Youngblood and Ferguson 2003; Wyka et al. 2007) and other evergreen species (Valladares et al. 2000). Especially the LMA, an integrative index of leaf structure known to be particularly sensitive to light conditions, showed relatively smaller differences in evergreen conifers. Larger LMA can be achieved by greater thickness of leaf lamina or tissue density, the latter involving an increased packing of cell wall and protoplast material, e.g., through increased wall sclerification or decreased fraction of intercellular spaces (Witkowski and Lamont 1991; Castro-Díez et al. 2000; Hassiotou et al. 2010). In interspecific comparisons, the relationship between LMA and leaf thickness is, however, frequently non-significant, unlike that between LMA and leaf density (Poorter et al. 2009). In contrast, if intraspecific variation was considered, the lower thickness clearly contributed to the decreased LMA in LL in both conifers and broadleaves. These results emphasize that light-related intraspecific variation in LMA may not result from the same underlying modifications as interspecific variation (Poorter et al. 2009).

The HL/LL differences in lamina thickness in broadleaves were clearly related to differences in thickness of palisade tissue. The lower plasticity in conifers and the less specialized anatomical processes underlying structural adjustment of their leaves, especially the conservative response of outermost palisade cells to light, may reflect phylogenetic constraints in this plant lineage (Lusk et al. 2003), perhaps related to the limited ability to form planar laminae by these single-veined leaves (Zwieniecki et al. 2004). Other factors that complicate the evaluation of adaptive value of mesophyll plasticity are the complex three-dimensional shape of conifer leaves and their pronounced clumping on the shoot, both features affecting light harvesting efficiency and subject to modification in shade (Niinemets 2010). Adjustment of shoot architecture might to some extent compensate for lower plasticity at the leaf and tissue level. Plasticity of leaf structure may also be limited by greater leaf longevity, because conifer leaves even in shade are designed to last for several years, and therefore biomass investment may be needed not only to construct photosynthetic tissue but also to ensure leaf durability, e.g., through greater tissue sclerification (Chabot and Hicks 1982; Castro-Díez et al. 2000) or tighter cell packing. Given the large volume fraction of support tissues in conifer foliage and the trade-off between mesophyll and structural tissues, it appears that plasticity in LMA and needle dimensions is indeed constrained by structural demands (Niinemets et al. 2007). This is supported by the report that, in evergreen angiosperms, shade leaves retained much of the mechanical strength of sun leaves, partly because of a conservative response of structural components (i.e. cell walls, as opposed to cell contents) to low light (Lusk et al. 2010).

LMA and its inverse, SLA (specific leaf area), have been shown to be robust indices of important ecophysiological traits, such as leaf longevity and mass-based photosynthetic rate in large multispecies datasets, including representatives of diverse life forms and habitats (Reich et al. 1997; Wright et al. 2004, 2005a, b). Whereas LMA is negatively related to *A*_max(mass)_, its relationship to *A*_max(area)_ is positive but weak, because a variety of leaf functional types differing in LMA may have similar *A*_max(area)_ (Wright et al. 2004, 2005a). We asked whether inclusion of leaf phenotypes produced by plastic responses to shade still supports these interspecific relationships. This was indeed the case for mass-based relationships when both groups were pooled; however, the shade-induced reduction of LMA did not affect *A*_max(mass)_ when either broadleaves or conifers were considered alone, pointing to the prevalence of differences between functional groups. While the pooled deciduous broadleaf versus evergreen conifer analysis yielded *A*_max(mass)_ versus LMA slope = −0.66, a corresponding slope given by Wright et al. (2005a) based on a larger global sample covering a broad range of species was as low as −0.94 for all trees in their dataset. This discrepancy was likely influenced by their inclusion of evergreen trees with large LMA exceeding those in our sample.

The LMA versus *A*_max(area)_ relationship in pooled data from both groups was weaker than in deciduous broadleaves alone, in agreement with the finding that determination coefficient increases when plant functional types are considered individually, especially in low-LMA species (Reich et al. 1998). In spite of the fact that *A*_max(area)_–LMA relationships in leaves sampled from different crown positions may be highly species-specific (Kazda et al. 2000), our broadleaf samples demonstrated a rather robust covariation of the two traits consistent with the fact that leaves in this category were structurally relatively uniform. In contrast, the connection between LMA and *A*_max(area)_ in conifers was probably weakened by the diversity of their internal structures, accommodating, for example, extensive secretory ducts and transfusion tissues. The stronger relationship between LMA and *A*_max(area)_ in broadleaves might also arise from larger contribution of chloroplast containing mesophyll tissue, especially the palisade, to leaf volume (and hence biomass).

Given the high nitrogen content of components of photosynthetic apparatus, the different contribution of specialized photosynthetic tissues to leaf volume might also influence the difference in whole-leaf N_mass_ between the functional types. The greater average N_mass_ in broadleaves likely explains the fact that trees in this group achieved similar *A*_max(area)_ rates as conifers in spite of their smaller LMA (Reich and Walters 1992; Reich et al. 1995, 1997). This is supported by the fact that *A*_max(area)_ versus LMA data for *P. peuce* needles (in Fig. 5) fell outside the core conifers and were aligned with broadleaves, probably reflecting their high N_mass_ level in spite of the high LMA. On the contrary, the greater accumulation of nitrogen in a given leaf area in conifers was not sufficient to produce a photosynthetic advantage of that group over broadleaves at comparable light availabilities because of a smaller PNUE in conifers. The overall lower PNUE in conifers is in agreement with lower allocation of nitrogen to photosynthesis in these and other evergreen leaves in which large nitrogen fraction may be present in the form of inactive rubisco or cell wall proteins, thus contributing little to leaf photosynthetic capacity (Lloyd et al. 1992; Warren and Adams 2004). However, the trade-off between cell wall and photosynthetic nitrogen was recently put into question, suggesting operation of additional nitrogen sinks (Harrison et al. 2009; Hikosaka and Shigeno 2009). Other reasons for lower PNUE in evergreen conifer leaves may include their greater CO_2_ diffusive resistance due to lower porosity, greater diffusion path length, and cell wall thickness (Syvertsen et al. 1995; Evans and von Caemmerer 1996; Hikosaka and Shigeno 2009). Restricted light penetration into thick evergreen leaves may further lower PNUE (Green and Kruger 2001). In contrast, given the specialized roles of palisade cells in ensuring flexible chloroplast dispatching and light transmission within the leaf (Terashima et al. 2006), the deciduous angiosperm leaf with its well-defined palisade tissue may well constitute a more efficient light utilization system, aided by its capacity for fine tuning of anatomical structure to ambient light levels. Considering all the above, differences in leaf anatomy appear to contribute in manifold ways to the less efficient utilization of leaf nitrogen by evergreen conifer foliage.

The positive relationship between leaf nitrogen and photosynthetic rate is fundamental for understanding and modeling canopy-level photosynthesis (Kull and Jarvis 1995; Hollinger 1996; Meir et al. 2002; Aranda et al. 2004). For several interspecific datasets, it has been shown that, on a mass basis, *A*_max_ is closely linked to N (Reich and Walters 1992) due to the fact that much of leaf nitrogen is used for construction of photosynthetic enzymes, especially rubisco (Björkman 1968). The slope of this relationship may vary according to plant functional group, leaf structural traits, and soil nutrient availability (Reich et al. 1994, 1995, 1998). In contrast, the relationship between *A*_max_ and N expressed on an area basis is usually less tight if significant at all (Reich and Walters 1992; Reich et al. 1994, 1999; Wright et al. 2004), especially if leaves vary in structure. However, such studies were predominantly based on interspecific variation in traits of HL leaves rather than on intra-canopy variability (Reich et al. 1995; Meir et al. 2002). Our approach combined the two sources of variability and demonstrated that mass-based *A*_max_-N relationship was actually stronger in a pooled dataset than in individual groups, whereas the area-based relationship was stronger when considered separately for conifers and broadleaves. Much unexplained variation in pooled area-based relationships is likely attributable to differences in photosynthetic constraints resulting from leaf structure as outlined above. The relationship between N_area_ and photosynthetic rate was tighter in broadleaves (Ellsworth and Reich 1993; Reich et al. 1995, 1998), whereas in conifers, HL and LL data points were much less co-linear. Especially, their LL leaves displayed a large dispersion and clearly reduced the predictive power of the otherwise robust area-based equation. In contrast, the mass-based relationship explained over 50% variation in *A*_max_ in a pooled sample, and its slope (1.88) was similar to that in a previously published large interspecific compilation of various life forms (1.42; Reich et al. 1999) despite the differences in sample composition. Thus, the reputedly universal interspecific relationship between *A*_max(mass)_ and N_mass_ was also supported by our results.

Robust mass-based photosynthetic relationships have been reported when, in addition to N, SLA or LMA was incorporated into the regression analysis (Reich et al. 1997, 1998; Wright et al. 2004). Multiple regression results for our two functional types considered separately yielded significant relationships that reflected bivariate analyses, and showed clearly that on mass basis, photosynthetic rate was predicted by nitrogen but not LMA, and LMA became important only when the low LMA broadleaves and high LMA conifer leaves were combined in the same dataset. Even then, N_mass_ explained the majority of variation in photosynthesis. Other studies reporting similar multi-species analyses (in which, however, HL leaves are preferentially sampled) show that the contributions of LMA (or SLA) and N_mass_ to determination of *A*_max(mass)_ are approximately equal (Table 2). This difference between our results and the published literature may be partly accounted for by the balanced inclusion of LL leaves in our dataset. When regression was run for HL data only, both slopes became more similar to each other and to those from published equations, also improving the determination coefficient (Table 2). Thus, while expressing photosynthetic potential on the basis of leaf chemical and structural properties accounts for much interspecific variation (Wright et al. 2004), the within-crown leaf variability adds another dimension to the issue of leaf economic spectra.

**Table 2:** Multiple regression statistics for the relationship between log(*A*_max(mass)_) and index of leaf structure (LMA or SLA) and N_mass_ in the current dataset and three published studies

Source	Components of regression equation		*n*	*r* ^2^	*P*
	Structure	Nitrogen			
This study, all data	−0.27 × log(LMA)	1.42 × log(N_mass_)	88	0.59	<0.001
This study, HL data only	−0.42 × log(LMA)	1.11 × log(N_mass_)	44	0.78	<0.001
Wright et al. (2004)	−0.57 × log(LMA)	0.74 × log(N_mass_)	706	0.63	<0.001
Reich et al. (1997)	0.71 × log(SLA)	0.77 × log(N_mass_)	104	0.85	<0.001
Reich et al. (1997)	0.82 × log(SLA)	0.88 × log(N_mass_)	109	0.80	<0.001
Reich et al. (1998)	0.78 × log(SLA)	0.84 × log(N_mass_)	213	0.86	<0.001

In literature sources, HL leaves were used preferentially but not exclusively. Since SLA is a reverse of LMA, respective slopes differ only in sign, and their absolute values may be directly compared

In summary, by studying common garden-grown trees representing diverse functional types, we found that adult evergreen conifer trees exhibit relatively smaller differentiation in leaf structural traits between extremes of canopy light gradient than deciduous broadleaf angiosperm trees. These differences likely reflect structural demands of conifer leaves resulting from their perennial life cycle, but also from ghosts of this lineage’s evolutionary past. We further demonstrated that leaf diversification in response to light gradient in these groups produces phenotypes that largely comply with rules established for HL leaves, although departures are greater in the evergreen leaves of conifers.

## Electronic supplementary material

Below is the link to the electronic supplementary material.

Supplementary material 1 (DOC 140 kb)
