# Supplementary material for: Responses of leaf structure and photosynthetic properties to intra-canopy light gradients: a common garden test with four broadleaf deciduous angiosperm and seven evergreen conifer tree species
Source: Oecologia. 2012 Feb 16;170(1):11–24. doi: 10.1007/s00442-012-2279-y (PMC3422461; doi:10.1007/s00442-012-2279-y)
Supplement: Supplementary file 1 — Supplementary material 1 (DOC 140 kb) [file 442_2012_2279_MOESM1_ESM.doc]

Responses of leaf structure and photosynthetic properties to intra-canopy light gradients: a common garden test with four broadleaf deciduous angiosperm and seven evergreen conifer tree species[[1]](#footnote-2)

Tomasz P. Wyka^1^, J. Oleksyn^2^, R. Żytkowiak^2^, P. Karolewski^2^, A.M. Jagodziński^2^, P.B. Reich^3^

^1^ Corresponding Author, Adam Mickiewicz University, Department of Biology, Institute of Experimental Biology, Laboratory of General Botany, Umultowska 89, 61-614 Poznań, Poland, [twyka@amu.edu.pl](mailto:twyka@amu.edu.pl)

^2^Polish Academy of Sciences, Institute of Dendrology, Parkowa 5, 62-035 Kórnik, Poland

^3^University of Minnesota, Department of Forest Resources, St. Paul, MN 55108, USA

**Online Resource 1.** Characterization of study plots: tree age, stand density, tree diameter at breast height (DBH), tree height, relative PPFD at the lowest branch. Means±S.E. were calculated jointly for each pair of plots, except in *Thuja plicata* where only one plot was sampled. For relative PPFD, means±S.E. are based on eight measurements (four per plot) per species.

| Species | Age  [years] | Stand density  [trees ha^-1^] | DBH  [cm] | Height  [m] | Relative PPFD at the lowest branch  [%] |
| --- | --- | --- | --- | --- | --- |
| Evergreen conifers |  |  |  |  |  |
| *Pinus peuce* | 40 - 48 | 1588 ± 313 | 19.0 ± 2.3 | 15.9 ± 0.8 | 6.9 ± 1.1 |
| *Abies cephalonica* | 61 | 729 ± 54 | 28.2 ± 0.1 | 19.6 ± 2.3 | 4.6 ± 0.7 |
| *Pseudotsuga menziesii* | 47 - 49 | 750 ± 50 | 28.4 ± 0.1 | 21.5 ± 0.1 | 6.6± 0.6 |
| *Abies grandis* | 49 - 61 | 502 ± 12 | 31.7 ± 0.6 | 24.4 ± 0.1 | 3.3 ± 0.3 |
| *Thuja plicata* | 44* | 1625* | 15.5* | 17.9* | 1.5* ± 0.2 |
| *Abies procera* | 38 - 42 | 1413 ± 138 | 19.9 ± 0.6 | 13.2 ± 0.5 | 9.7 ± 0.7 |
| *Chamaecyparis pisifera* | 61 | 1163 ± 113 | 22.3 ± 0.1 | 18.1 ± 0.5 | 6.0 ± 0.2 |
| Deciduous Broadleaves |  |  |  |  |  |
| *Acer saccharum* | 37 | 1062 ± 123 | 14.5 ± 0.8 | 14.1 ± 0.9 | 2.8 ± 0.2 |
| *Acer rubrum* | 39 | 1064 ± 13 | 18.0 ± 0.2 | 20.7 ± 0.0 | 2.6 ± 0.1 |
| *Betula alleghaniensis* | 36 | 1151 ± 0 | 15.7 ± 0.2 | 13.7 ± 0.2 | 6.9 ± 0.5 |
| *Quercus rubra* | 52 - 61 | 413 ± 88 | 32.3 ± 1.0 | 23.8 ± 2.5 | 5.6 ± 0.4 |
|  |  |  |  |  |  |

* data for a single plot

**Online Resource 2.** Climate and Soil Characteritics

Climate of the region is transitional between maritime and continental, and the average annual precipitation is 596 mm (ranging between 404 and 832 mm) with about 70% falling between May and August. Average temperature was 7.2 °C with a mean growing season of about 212 days, calculated as the number of days with an average temperature ≥ 5 °C (weather data recorded for 55 years, ca. 1.5 km from the research area; Jagodzinski and Banaszczak 2010). Prior to the establishment of the experiment the site was a mature Scots pine (*Pinus sylvestris* L.) dominated forest. The soils were developed on a postglacial formation, in the region of a ground moraine. They represent the grey-brown podzolic type with horizons O-A-Eet-Bt-C. The pH is 5.0 in the humus layer, 4.2 in the topsoil and 4.8 in the eluvial horizon (Czępińska-Kamińska et al. 1991, unpublished manuscript available at the Rogow Arboretum, Poland).

Reference

Jagodzinski AM, Banaszczak P (2010) Stem volume and aboveground woody biomass in noble fir (*Abies procera* Redher) stands in the Rogów Arboretum (Poland). *Acta Sci Pol, Silvarum Colendarum Ratio et Industria Lignaria,* 9:9-24

**Online Resource 3**. Structural and photosynthetic trait values (means ± S.E.) for leaves from fully illuminated (HL) and most shaded (LL) locations within the crown in seven evergreen conifer and four broadleaf deciduous angiosperm species. Significance values for ANOVA effects are shown separately for each taxonomic group and for the entire sample (* *P*<0.05, ** *P*<0.01, *** *P*<0.001, n.s. effect not significant). Significant (*P*<0.05) contrasts between HL and LL leaves within each species are indicated by bold font.

| Traits | Light | *Pinus*  *peuce* | *Abies*  *cephalonica* | *Pseudotsuga menziesii* | *Abies*  *grandis* | *Thuja*  *plicata* | *Abies*  *procera* | *Chamaecyparis pisifera* | ANOVA effects  Conifers | | | *Acer saccharum* | *Acer rubrum* | *Betula alleghanensis* | *Quercus rubra* | ANOVA effects  Broadleaves | | | ANOVA effects  All species | | |
| --- | --- | --- | --- | --- | --- | --- | --- | --- | --- | --- | --- | --- | --- | --- | --- | --- | --- | --- | --- | --- | --- |
|  |  |  |  |  |  |  |  |  | Species | Light | Species  × Light |  |  |  |  | Species | Light | Species  × Light | Species | Light | Species  × Light |
| STRUCTURAL |  |  |  |  |  |  |  |  |  |  |  |  |  |  |  |  |  |  |  |  |  |
| LMA (g m^-2^) | HL | **171.7 ± 5.0** | **171.8 ± 4.0** | **172.0 ±16.2** | **173.3 ±7.0** | **180.9 ± 13.4** | **217.0 ± 16.9** | **228.4 ± 15.5** | * | *** | * | **51.9 ± 10.1** | **61.9 ± 6.9** | **63.3 ± 2.2** | **72.8 ± 12.4** | ** | *** | n.s. | *** | *** | *** |
|  | LL | **129.2 ± 2.8** | **138.1 ± 3.5** | **118.1 ±1.8** | **118.6 ±8.2** | **119.0 ± 6.3** | **146.4 ± 18.5** | **112.0 ±7.8** |  |  |  | **23.5 ± 1.5** | **30.5 ± 1.8** | **28.7 ± 1.8** | **43.0 ± 4.0** |  |  |  |  |  |  |
|  |  |  |  |  |  |  |  |  |  |  |  |  |  |  |  |  |  |  |  |  |  |
| Leaf tissue density  (g cm^-3^) | HL | 0.408 ± 0.012 | **0.324 ± 0.008** | 0.379 ± 0.011 | **0.383 ± 0.018** | **0.244 ± 0.023** | **0.516 ± 0.009** | **0.375 ± 0.05** | *** | n.s. | *** | **0.539 ± 0.009** | **0.513 ± 0.020** | **0.477 ± 0.012** | 0.495 ± 0.031 | *** | *** | *** | *** | *** | *** |
|  | LL | 0.399 ± 0.011 | **0.404 ± 0.009** | 0.328 ± 0.021 | **0.308 ± 0.015** | **0.299 ± 0.022** | **0.433 ± 0.011** | **0.256 ± 0.023** |  |  |  | **0.317 ± 0.010** | **0.428 ± 0.009** | **0.280 ± 0.021** | 0.536 ± 0.011 |  |  |  |  |  |  |
|  |  |  |  |  |  |  |  |  |  |  |  |  |  |  |  |  |  |  |  |  |  |
| Lamina thickness  (μm) | HL | **707.3 ± 3.0** | **569.9 ± 11.7** | **527.8 ±12.5** | **519.7 ±20.3** | **533.6 ± 6.5** | **464.9 ±33.01** | **793.87 ± 59.0** | *** | *** | n.s. | **93.0 ± 1.4** | **126.3 ± 4.9** | **132.5 ± 3.3** | **149.4 ± 8.0** | *** | *** | *** | *** | *** | *** |
|  | LL | **547.7 ± 11.0** | **380.4 ± 4.4** | **416.4 ± 28.4** | **427.9 ±18.4** | **446.0 ± 30.8** | **395.2 ±14.36** | **550.5 ± 31.2** |  |  |  | **74.1 ± 1.4** | **72.7 ± 2.3** | **102.8 ± 3.3** | **78.6 ± 0.7** |  |  |  |  |  |  |
|  |  |  |  |  |  |  |  |  |  |  |  |  |  |  |  |  |  |  |  |  |  |
| Total mesophyll  thickness (μm) | HL | **350.7 ± 9.0** | **363.6 ± 4.6** | **367.0 ± 17.5** | **358.4 ± 20.9** | **483.9 ± 10.9** | **351.5 ± 12.1** | **658.2 ± 84.5** | *** | *** | n.s. | **69.8 ± 1.7** | **94.6 ± 6.0** | **101.1 ± 0.6** | **127.1 ± 9.7** | *** | *** | *** | *** | *** | *** |
|  | LL | **250.1 ± 19.1** | **246.9 ± 7.1** | **299.5 ± 20.8** | **289.8 ± 20.6** | **406.0 ± 25.7** | **280.9 ± 23.6** | **472.1 ± 26.4** |  |  |  | **55.0 ± 1.6** | **50.2 ± 2.5** | **77.7 ± 2.4** | **55.2 ± 1.0** |  |  |  |  |  |  |
|  |  |  |  |  |  |  |  |  |  |  |  |  |  |  |  |  |  |  |  |  |  |
| Palisade thickness  (μm) | HL | 100.7 ± 17.7 | **36.9 ± 1.7** | **36.8 ± 4.3** | **38.1 ±3.0** | 101.3 ± 2.8 | **25.1 ± 1.7** | **18.2 ± 4.5** | *** | * | * | **42.9 ± 1.2** | **56.0 ± 3.4** | **44.9 ± 1.3** | **90.1 ± 4.4** | *** | *** | *** | *** | *** | *** |
|  | LL | 92.3 ± 8.9 | **26.0 ± 0.8** | **23.0 ± 3.7** | **24.0 ±3.4** | 86.2 ± 9.8 | **46.3 ± 17.2** | **11.0 ± 1.6** |  |  |  | **30.1 ± 0.3** | **24.6 ± 0.3** | **31.7 ± 1.1** | **25.9 ± 0.8** |  |  |  |  |  |  |
|  |  |  |  |  |  |  |  |  |  |  |  |  |  |  |  |  |  |  |  |  |  |
| Outer palisade  cell length (μm) | HL | 48.4 ± 7.7 | **89.3 ± 3.4** | 51.6 ± 5.8 | 66.2 ± 3.7 | 56.8 ±0.6 | 44.9 ± 4.5 | 52.7 ±5.6 | *** | n.s. | n.s. | **42.9 ± 1.2** | **56.0 ± 3.4** | **22.8 ± 0.7** | **37.9 ± 1.1** | *** | *** | *** | *** | *** | *** |
|  | LL | 56.0 ± 7.1 | **66.4 ± 10.3** | 41.0 ± 7.2 | 71.6 ± 4.9 | 60.3 ±2.8 | 49.3 ± 7.1 | 44.9 ±6.4 |  |  |  | **30.1 ± 0.3** | **24.6 ± 0.3** | **31.7 ± 1.1** | **25.9 ± 0.8** |  |  |  |  |  |  |
|  |  |  |  |  |  |  |  |  |  |  |  |  |  |  |  |  |  |  |  |  |  |
| Palisade/  mesophyll ratio | HL | **0.28 ± 0.05** | 0.10 ± 0.00 | 0.10 ± 0.02 | 0.11 ± 0.02 | 0.21 ± 0.01 | **0.07 ± 0.01** | 0.03 ± 0.01 | *** | n.s. | ***** | **0.61 ± 0.02** | **0.59 ± 0.02** | 0.44 ± 0.02 | **0.71 ± 0.02** | *** | *** | *** | *** | ** | *** |
|  | LL | **0.37 ± 0.02** | 0.11 ± 0.00 | 0.08 ± 0.01 | 0.09 ± 0.02 | 0.21 ± 0.02 | **0.15 ± 0.04** | 0.02 ± 0.00 |  |  |  | **0.55 ± 0.01** | **0.49 ± 0.02** | 0.41 ± 0.02 | **0.47 ± 0.02** |  |  |  |  |  |  |
|  |  |  |  |  |  |  |  |  |  |  |  |  |  |  |  |  |  |  |  |  |  |
| PHOTOSYNTHETIC |  |  |  |  |  |  |  |  |  |  |  |  |  |  |  |  |  |  |  |  |  |
|  |  |  |  |  |  |  |  |  |  |  |  |  |  |  |  |  |  |  |  |  |  |
| N_area_ (g m^-2^) | HL | 3.25 ± 0.50 | 2.09 ± 0.17 | **2.35 ± 0.24** | **2.12 ± 0.18** | **2.43 ± 0.26** | **3.02 ± 0.48** | **2.92 ± 0.17** | *** | *** | n.s. | **0.98 ± 0.09** | **1.12 ± 0.04** | **1.39 ± 0.04** | **1.71 ± 0.27** | *** | *** | n.s. | *** | *** | ** |
|  | LL | 2.65 ± 0.36 | 1.80 ± 0.10 | **1.51 ± 0.03** | **1.39 ± 0.12** | **1.48 ± 0.12** | **1.58 ± 0.15** | **1.74 ± 0.06** |  |  |  | **0.52 ± 0.02** | **0.57 ± 0.05** | **0.58 ± 0.04** | **0.86 ± 0.14** |  |  |  |  |  |  |
|  |  |  |  |  |  |  |  |  |  |  |  |  |  |  |  |  |  |  |  |  |  |
| N_mass_ (% d.m.) | HL | 1.89 ± 0.25 | 1.21 ± 0.08 | 1.37 ± 0.08 | 1.23 ± 0.12 | 1.34 ± 0.10 | 1.37 ± 0.12 | **1.29 ±0.09** | *** | n.s. | n.s. | 1.98 ± 0.19 | 1.87 ± 0.17 | 2.20 ± 0.14 | 2.37 ±0.16 | n.s. | n.s. | n.s. | *** | n.s. | n.s. |
|  | LL | 2.03 ± 0.23 | 1.31 ± 0.07 | 1.28 ± 0.02 | 1.17 ± 0.04 | 1.24 ± 0.05 | 1.10 ± 0.08 | **1.58 ± 0.13** |  |  |  | 2.21 ±0.07 | 1.86 ± 0.11 | 2.02 ± 0.07 | 1.98 ±0.17 |  |  |  |  |  |  |
|  |  |  |  |  |  |  |  |  |  |  |  |  |  |  |  |  |  |  |  |  |  |
| A_max(area)_  (μmol m^-2^ s^-1^) | HL | **16.02 ± 0.99** | **6.69 ± 1.34** | 8.00 ± 1.27 | 8.38 ± 0.48 | **5.56 ± 0.84** | **10.23 ± 0.96** | **7.84 ± 0.71** | *** | *** | n.s. | 5.60 ± 0.94 | **6.99 ± 0.51** | **7.72 ± 0.71** | **10.80 ± 0.92** | ** | *** | n.s. | *** | *** | n.s. |
|  | LL | **13.69 ± 0.37** | **4.25 ± 0.98** | 6.51 ± 0.17 | 6.49 ± 0.40 | **2.72 ± 0.88** | **4.65 ± 1.13** | **3.15 ± 0.37** |  |  |  | 3.97 ± 0.26 | **3.23 ± 0.78** | **2.84 ± 0.40** | **5.56 ± 1.02** |  |  |  |  |  |  |
|  |  |  |  |  |  |  |  |  |  |  |  |  |  |  |  |  |  |  |  |  |  |
| A_max(mass)_  (nmol g^-1^ s^-1^) | HL | 93.11 ± 3.47 | 38.72 ± 7.20 | 46.45 ± 5.56 | 48.57 ± 3.18 | 30.58 ± 3.28 | 47.28 ± 2.90 | 34.61 ± 3.33 | *** | n.s. | n.s. | **110.0 ± 9.8** | 118.6 ± 19.0 | 121.2 ± 6.8 | 155.8 ± 15.7 | n.s. | n.s. | * | *** | n.s. | * |
|  | LL | 106.21 ± 4.50 | 30.39 ± 6.49 | 55.17 ± 1.81 | 54.91 ± 2.19 | 23.15 ± 7.36 | 34.83 ± 10.24 | 28.61 ± 4.14 |  |  |  | **172.3 ± 19.2** | 104.1 ± 20.9 | 98.3 ± 11.2 | 126.4 ±10.7 |  |  |  |  |  |  |
|  |  |  |  |  |  |  |  |  |  |  |  |  |  |  |  |  |  |  |  |  |  |
| PNUE  (μmol gN^-1^ s^-1^) | HL | 5.12 ± 0.44 | 3.11 ± 0.38 | 3.45 ± 0.47 | 4.01 ± 0.30 | 2.35 ± 0.37 | 3.49 ± 0.24 | 2.72 ± 0.31 | *** | n.s. | n.s. | **5.62 ± 0.37** | 6.24 ± 0.48 | 5.43 ± 0.63 | 6.64 ± 0.77 | n.s. | n.s. | n.s. | *** | n.s. | n.s. |
|  | LL | 5.48 ± 0.10 | 2.39 ± 0.57 | 4.32 ± 0.10 | 4.70 ± 0.15 | 1.91 ± 0.64 | 3.10 ± 0.87 | 1.82 ± 0.22 |  |  |  | **7.74 ± 0.67** | 5.68 ± 1.26 | 4.84 ± 0.43 | 6.45 ± 0.56 |  |  |  |  |  |  |

1. [↑](#footnote-ref-2)
